# Implementation and feasibility of DPYD-guided fluoropyrimidine use in a gastrointestinal oncology clinic at an academic medical center

**DOI:** 10.3389/fonc.2026.1944703

**Published:** 2026-09-30

**Authors:** Madeline L. Norris, Khoa A. Nguyen, Emily J. Cicali, Wandyeind Marquez Moyeno, Je-Won J. Hong, Katherine Vogel Anderson, Tim Jang, Gahyun Gim, Kathleen Sullivan, Alexandra Kennan, Petr Starostik, Thomas J. George, David L. DeRemer, Larisa H. Cavallari, Julio D. Duarte

**Affiliations:** 1 Department of Pharmacotherapy and Translational Research, College of Pharmacy, University of Florida, Gainesville, FL, United States; 2 Center for Pharmacogenomics and Precision Medicine, College of Pharmacy, University of Florida, Gainesville, FL, United States; 3 University of Florida Health Cancer Institute, Gainesville, Florida, FL, United States; 4 Division of Hematology and Oncology, Department of Medicine, College of Medicine, University of Florida, Gainesville, FL, United States; 5 Department of Pathology, Immunology, and Laboratory Medicine, College of Medicine, University of Florida, Gainesville, FL, United States

**Keywords:** DPYD, feasibility, fluoropyrimidines, implementation, pharmacogenetics, pharmacogenomics

## Abstract

**Introduction:**

Genetic variation in *DPYD* can result in reduced fluoropyrimidine (FP) metabolism and increased risk of severe toxicity. This *DPYD* test implementation pilot aimed to establish a feasible workflow for *DPYD* ordering sample collection and FP dose modifications at the University of Florida Health Cancer Institute.

**Patients and methods:**

Following education on the clinical utility of DPYD testing, a consensus was reached with the University of Florida Health gastrointestinal oncology clinic on an implementation workflow. Novel clinical decision support (CDS) tools were developed including pre- and post-test inline warnings in chemotherapy treatment plans, clickable dose modification buttons, and a pharmacist-facing alert upon medication verification. Primary outcomes were feasibility metrics including rates and timing of test ordering and sample collection, genotype turnaround time, and clinician response to CDS tools (e.g., FP dose modifications).

**Results:**

*DPYD* was ordered for 201 patients between July 2024 and March 2026. Most (180/201) orders were placed prior to chemotherapy in FP-naïve individuals, and 85% (153/180) were also collected pretreatment. The average genotype turnaround time was 5.0 ± 2.7 days. Results were available prior to chemotherapy for 80.3% (114/142) of all patients who received a FP-containing regimen and for 93.2% (110/118) of those with DPYD ordered and collected pretreatment. Nine patients had a deleterious DPYD variant; all who newly initiated a FP-containing regimen received a 50% dose reduction.

**Discussion:**

DPYD test implementation with customized CDS tools to support test ordering and FP prescribing in an academic oncology clinic is feasible, with the majority of results returning prior to chemotherapy. Integrating new and unique processes into already established workflows helps achieve timely test ordering and sample collection.

## 1 Introduction

Fluoropyrimidines (FP) are a class of chemotherapy drugs utilized to treat a variety of solid tumors, including but not limited to, colorectal, pancreatic, breast, bladder, and head and neck cancers (1–3). 5-fluorouracil (5-FU) is an intravenous chemotherapy agent used in combination with other chemotherapy agents, while capecitabine is an oral prodrug that is converted to 5-FU in the liver. In the United States (US) alone, it is estimated that over 250,000 patients receive a (FP) annually (4, 5). While these drugs are considered a cornerstone of therapy, they are also associated with potentially life-threatening toxicity (e.g., diarrhea, stomatitis, hand-foot syndrome, and neutropenia) (6–8). It is estimated that more than 1,300 patients in the US die each year as a result of FP toxicity (5). The occurrence of these severe toxicities is estimated at 20-30% overall and between 60-80% for patients who have a deleterious variant in *DPYD* (9–11). *DPYD* encodes for dihydropyrimidine dehydrogenase (DPD), the rate-limiting enzyme responsible for FP catabolism (12). Individuals who have reduced DPD enzymatic activity are at risk for reduced FP clearance and increased exposure to the drug, which increases the risk of toxicity and death (10, 13–16).

To mitigate toxicity, international guidelines recommend avoiding or reducing the dose of FPs based on the degree of DPD deficiency (DPD phenotype) (17, 18). The Clinical Pharmacogenomics Implementation Consortium (CPIC) recommends utilizing a *DPYD* activity score, calculated based on the diplotype, to predict DPD phenotype. An activity score of 0 or 0.5 is defined as a poor metabolizer (PM), 1 or 1.5 designates an intermediate metabolizer (IM), and a score of 2.0 is defined as a normal metabolizer (NM) (19). CPIC recommends a 50% reduction in dose for IMs and to avoid FP use in PMs. An important consideration for IMs who receive an initial dose reduction is to utilize dose escalation based on tolerability in subsequent FP cycles. This strategy accounts for the range of maximum tolerated doses that exist among patients with partial DPD deficiency, and is critical to avoid negative treatment outcomes (i.e., poor progression-free survival and overall survival) from dose reductions as demonstrated by Knikman et al. (20).

Despite strong evidence supporting *DPYD*-guided FP treatment, adoption has been slow in the US. In contrast, *DPYD* testing is the standard of care among many European countries, with the European Medicine Agency (EMA) recommending pre-treatment DPD testing since 2020 (21). While the US Food and Drug Administration (FDA)-approved FP labeling has acknowledged the relationship between DPD deficiency and toxicity risk, it was not until October 2025 that a boxed warning was added regarding adverse reactions in patients with a complete DPD deficiency and recommending that patients undergo *DPYD* genotyping prior to initiating capecitabine or fluorouracil unless immediate treatment is necessary” (22, 23). The FP labeling update also acknowledges the need to individualize dosing in patients with partial DPD deficiency (IMs), although no specific dose recommendations were provided. Accordingly, the National Comprehensive Cancer Network (NCCN) updated the colon cancer guidelines to recommend testing and to avoid capecitabine and 5-FU in patients with complete DPD deficiency (1). These updates are expected to expedite implementation efforts of *DPYD*-guided FP treatment in the US. Several early adopters of DPYD testing have described their implementations processes and feasibility metrics (13, 24–27).

The University of Florida (UF) Health Cancer Institute is a NCI-Designated Clinical Cancer Center. Considering the high utilization of FP-based regimens in GI cancers, the UF Health Cancer Institute’s gastrointestinal (GI) oncology clinic was the most logical place to begin the implementation of *DPYD*-guided 5-FU and capecitabine use at our institution. We piloted implementing in-house *DPYD* testing in the UF Health Cancer Institute as part of a quality improvement project, which aimed to establish the feasibility of *DPYD*-guided FP use. Herein we describe our implementation efforts, feasibility metrics, challenges experienced, and lessons learned.

## 2 Patients and methods

To facilitate initial engagement from the oncology team education on the evidence supporting the clinical utility of *DPYD* was provided. The information presented included a description of the relationship between DPD deficiency and FP exposure, risk of FP-associated adverse effects and clinical outcome data showing no significant difference between patients who received *DPYD*-guided standard FP dosing. Further discussions related to the GI oncology clinic’s workflow resulted in an initial consensus on the process for genotype ordering and sample collection, acceptable dose recommendations, and the clinical decision support (CDS) tools that would be developed, each of which are described in detail below. Feasibility metrics including number of tests ordered, rate and timing of sample collection, test turnaround time, and response to CDS tools were collected throughout the pilot period (July 2024-March 2026).

### 2.1 Pre-test workflow

#### 2.1.1 Patient identification and education

The UF Health Cancer Institute GI oncology clinic schedule was screened weekly by a hematology/oncology fellow for new patients likely to receive a FP-containing regimen (e.g., FOLFOX, CAPOX) based on clinical judgement. Previous use of any FP was also reviewed to limit testing to FP-naive individuals in whom response cannot be predicted from prior use. If the patient fit these criteria, a standardized appointment note was added into their scheduled clinic visit in the electronic health record (EHR). The appointment note stated that the patient was a potential candidate for *DPYD* pharmacogenetic (PGx) testing and instructed clinic staff to (i) verify that an order for the test had been placed, and (ii) if ordered, ensure a DNA sample was collected. Instructions were also included for sample collection and labeling. Considering *DPYD* genotyping had not historically been available at our institution, this screening process and appointment note served as an aid to identify appropriate patients in the early phases of the pilot and as a reminder to clinicians to encourage testing until doing so became a more standard part of practice. The decision for testing was still ultimately provided by the treating oncologist.

To support patient engagement and shared decision-making, pre-test patient-facing educational materials that are accessible, relevant and aligned with patient needs were developed for distribution in clinic (**Figure 1**).

**Figure 1:**
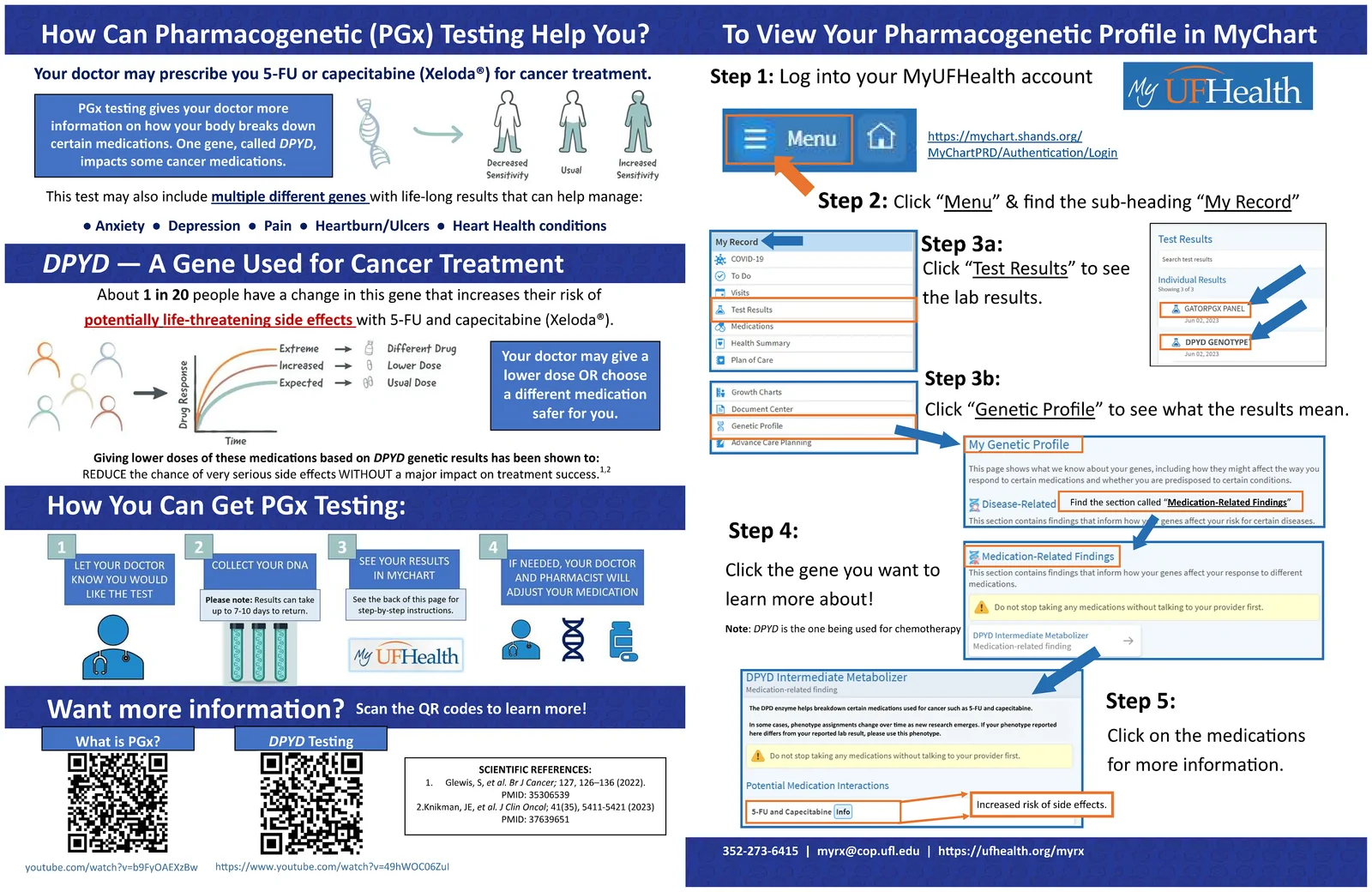
University of Florida (UF) Health pretest educational patient handout (© 2026 Epic Systems Corporation).

#### 2.1.2 Genotyping and sample collection

A total of eight decreased or no function *DPYD* variants were included on the UF Health *DPYD* genotyping panel developed internally: *2A, *13, HapB3(c.1129–5923C>G, c.1236G>A), c.2846A>T, c.557A>G, c.1024G>A, c.1774C>T, c. 2279C>T. Clinical genotyping was performed using PCR-based TaqMan^®^ SNP Genotyping Assays on an Applied Biosystems QuantStudio (Life Technologies) by UF Health Molecular Pathology Laboratory. Results were reported into the patient’s EHR as discrete variables, similar to other clinical PGx testing completed at UF Health.

Test turnaround time (from the laboratory receiving the sample to resulting) was estimated to be 7–10 days, consistent with the estimated number of days between initial chemotherapy ordering and patient infusion appointment. Thus, early sample collection was a priority of our implementation effort to maximize the clinical utility of PGx testing. The decision to move forward with chemotherapy is typically made at the first clinic visit following cancer diagnosis, hence, this was identified as the earliest potential time to collect a DNA sample for PGx testing. Providers were encouraged to order *DPYD* if they anticipated the patient would receive a FP containing regimen, even if a final chemotherapy decision was not yet made.

Buccal swab collection kits were stocked in clinic for sample collection by nursing staff during the clinic appointment. Four cheek swab labels were configured to print automatically after the “collect” action was acknowledged on an order in the EHR. In-clinic buccal swabs were proposed to ensure timely DNA sample collection in the event that patients did not have their blood drawn at the initial clinic visit. Blood draws were favored by some clinic staff in scenarios where blood was already being collected for other purposes, thus, workflows were developed for the collection of either sample type to best meet the preferred workflow for the individual patient.

#### 2.1.3 Clinical decision support

Computerized CDS (28) plays an essential role in assisting healthcare providers order and interpret *DPYD* tests and their results (29). Initially, we embedded the PGx-test recommendations directly into cycle 0 of the treatment plan to remind providers to order a PGx test (**Figure 2a**). This communication required manually opening the “Provider Communication” tab to view, a step that may have easily been missed upon entering a treatment plan. We subsequently have added an inline medication warning (an Epic alert type that embeds recommendations directly below the medication order) to inform providers about PGx testing (**Figure 2b**).

**Figure 2:**
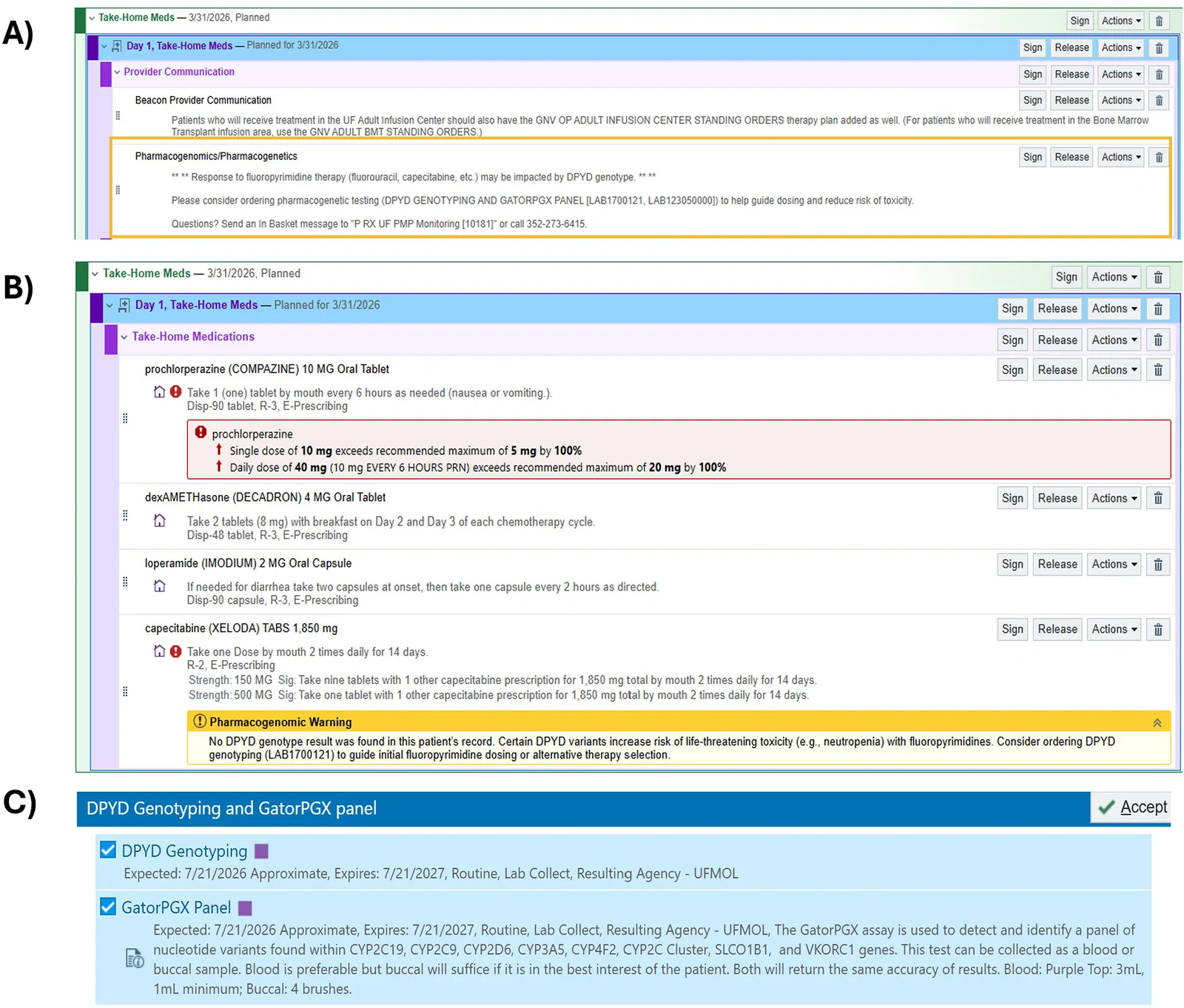
UF Health EHR pretest CDS for *DPYD* test ordering (^©^ 2026 Epic Systems Corporation). **(A)** Pretest recommendation embedded directly in care plan **(B)** Pretest in-line recommendation embedded directly in medication **(C)** Available order set for provider to pick desired PGx test(s).

To further support pretreatment genotyping and streamline clinical workflows, a dedicated order set was developed encompassing both targeted *DPYD* testing and broader PGx panel option to help guide supportive care prescribing (**Figure 2c**). Embedding these options within a structured order set reduces variability in ordering practices and reinforces consistent application of *DPYD* testing prior to FP order. Specific metrics related to the multi-gene PGx panel for supportive care will be described separately from this paper.

### 2.2 Post-test workflow

Once PGx results were available, they were automatically linked to the patient’s chart as discrete test result variables using HL7 standards with associated genomic indicators (Epic’s version of genomics phenotypes) to integrate PGx data into Epic’s Genomics Module, a data model and implementation process described in detail by Newsom et al. (2024) (30). The use of genomic indicators enhances the clinical utility of PGx data by enabling alert logic and supporting effective delivery of phenotype-informed recommendations to providers at the point of care, including within medication orders. For patients with a documented *DPYD* phenotype, activity score-specific dose reduction options are presented as clickable selections in the treatment plan (**Figure 3a**), each displaying the recommended percent dose reduction and linking directly to the relevant CPIC guideline (17) with detailed dosing guidance (**Figure 3b**). Post-test inline warnings embedded within each cycle of the treatment plan also include specific recommendations for the patient based on their results, including a reminder to titrate reduced doses upward based on patient tolerability (**Figure 4**). This design is particularly valuable in the oncology setting, where multi-cycle regimens span weeks to months and the risk of drug-gene interactions being overlooked increases over time without persistent reminders.

**Figure 3:**
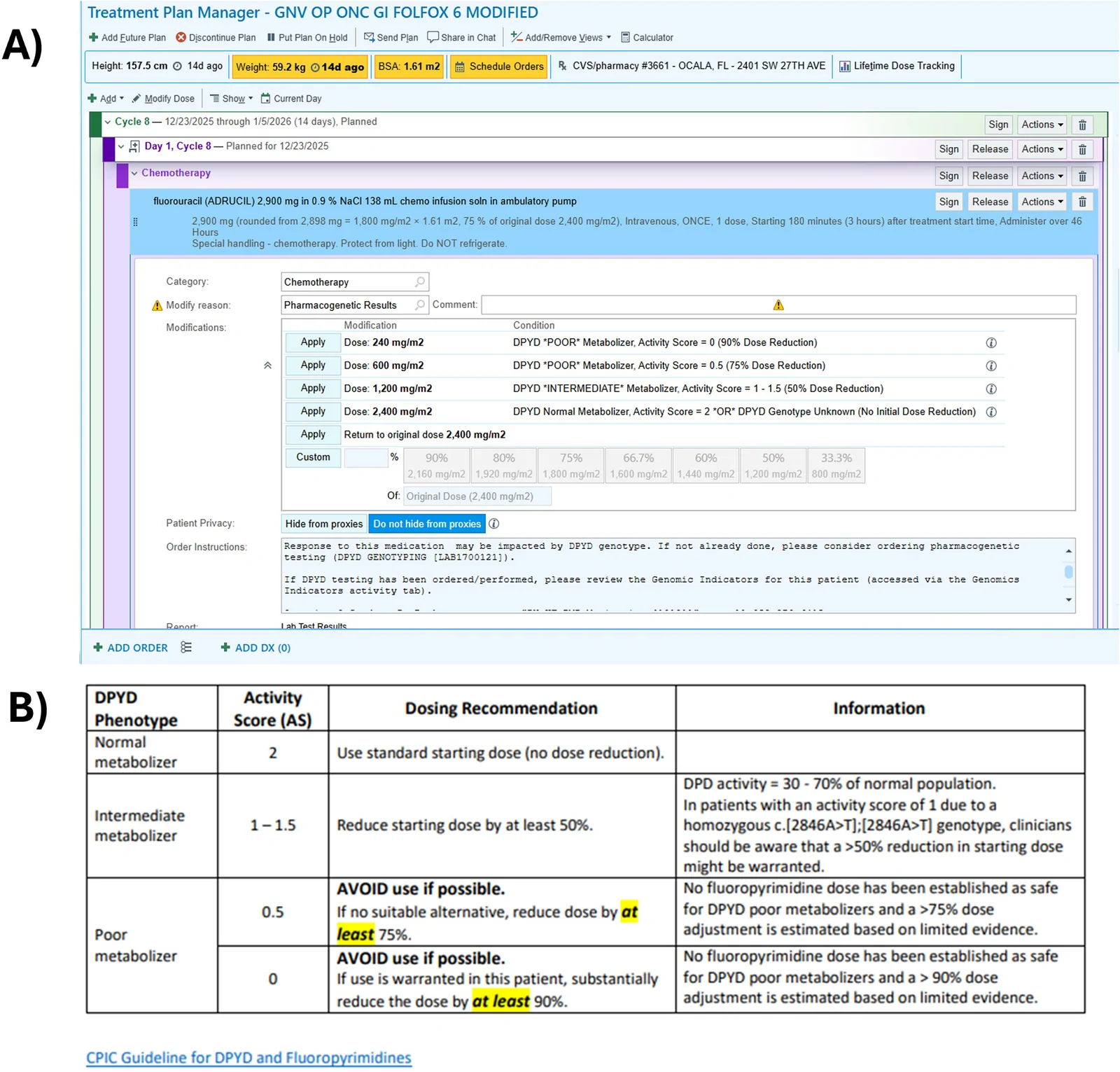
UF Health EHR dose adjustment guidance (^©^ 2026 Epic Systems Corporation). **(A)** Adjustable buttons for fluorouracil dosing in treatment plan and **(B)** CPIC guideline reference table for *DPYD*-guided fluoropyrimidine use.

**Figure 4:**
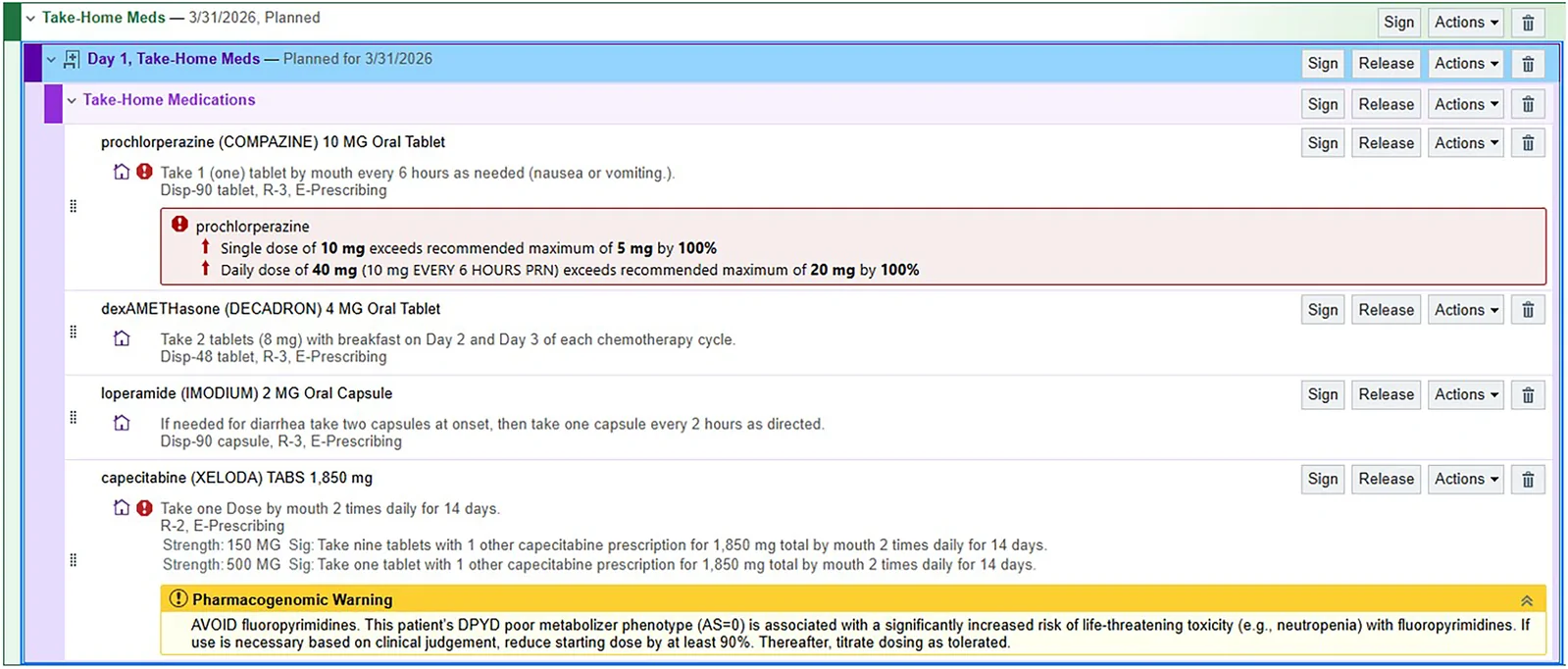
UF Health EHR (^©^ 2026 Epic Systems Corporation) post-test inline medication warning embedded in treatment plan.

Pharmacist-facing alerts were implemented as an additional safeguard within the *DPYD*-guided FP workflow (**Figure 5**). These alerts were built using Epic’s native pharmacogenomics Our Practice Alert (OPA) framework, with the trigger configured at the point of medication verification prior to 5-FU infusion or when capecitabine prescriptions are received by the UF Health specialty pharmacy. Capecitabine orders filled outside of UF Health currently rely on the provider-facing CDS described above. This represents the first pharmacist-facing PGx OPA implemented at our institution. *DPYD* activity score-specific recommendations mirroring those presented to prescribers are delivered to pharmacists at the verification step, reinforcing genotype-informed dose selection and serving as a critical final checkpoint before medication dispensing.

**Figure 5:**
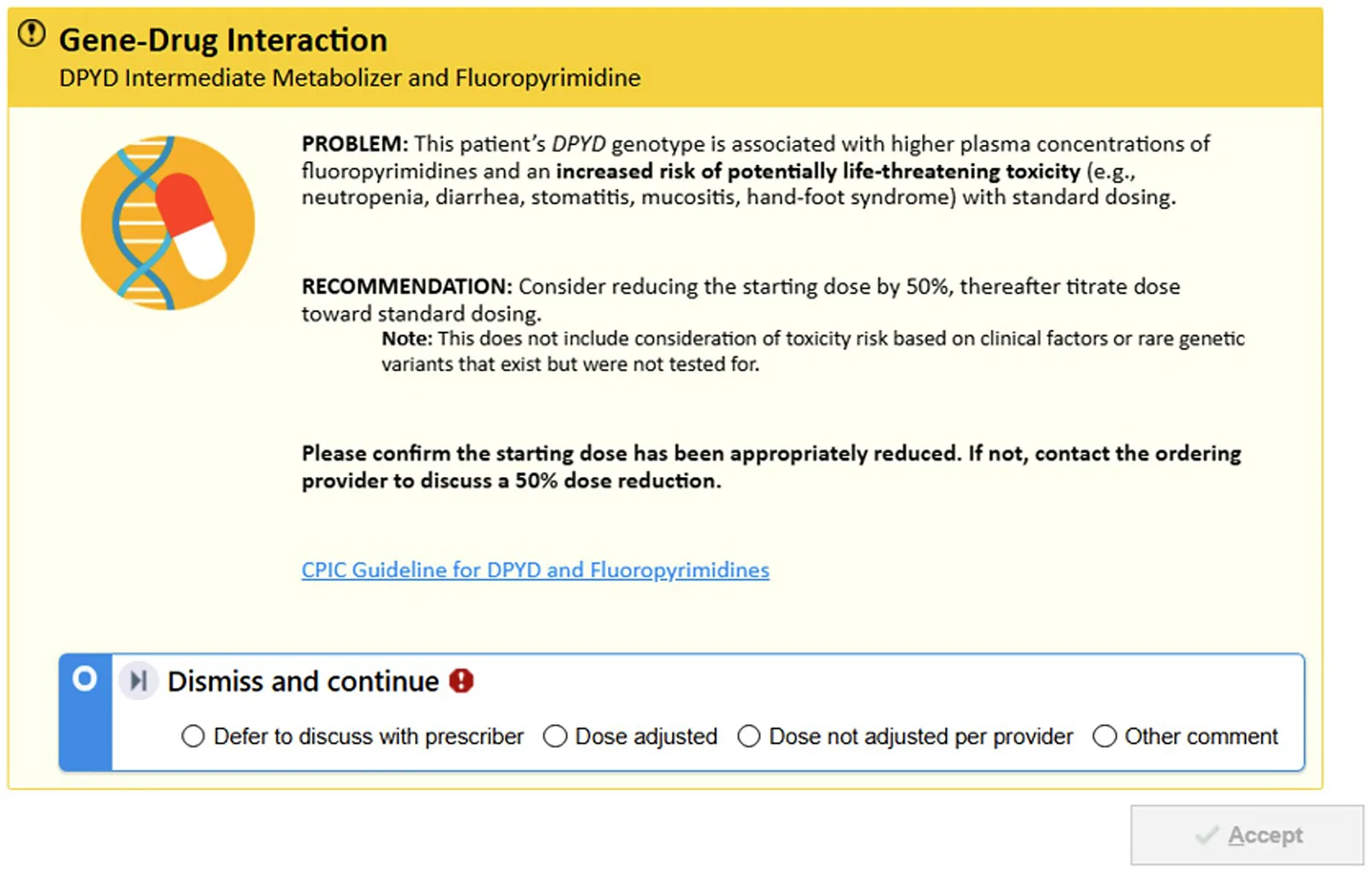
Pharmacist-facing post-test our practice advisory (OPA) alert for a *DPYD* intermediate metabolizer at time of medication verification (^©^ 2026 Epic Systems Corporation).

In addition to automated CDS, clinical consult notes with genotype-specific interpretations and medication recommendations were provided by a PGx pharmacist for all *DPYD* variant carriers and stored in the EHR. In the case that a PM was identified, the treating oncologist, nurse, and pharmacy staff would also be immediately notified via direct message in the Epic secure chat environment. All FP recommendations were personalized to the patient’s planned chemotherapy regimen (e.g., mFOLFOX6, FOLFIRI) if available but remained consistent with institutional guidance developed based on CPIC guidelines (**Figure 3b**). Similar to guidance included in the treatment plan, an upward dose titration based on tolerability was recommended in consult notes for IMs.

## 3 Results

### 3.1 Patient genotyping and sample collection

*DPYD* genotyping was ordered for 201 patients, 89.5% (180/201) of which were ordered at least one day prior to treatment in FP-naïve patients (i.e., pretreatment). Most patients were female and self-reported as White and non-Hispanic/Latino, with a mean age of 62 ± 11 years old (**Table 1**). Observed demographics in this pilot were similar to the approximate demographics of the adult oncology clinic overall. The most common cancer types were rectal, colon and pancreatic. A DNA sample was collected (via blood draw or buccal swab) for 90% (181/201) of the total *DPYD* test orders. Of the orders placed pretreatment, 8.3% (15/180) were not collected and 6.7% (12/180) were collected the same day as or after chemotherapy initiation (**Table 1**). All 181 samples were successfully genotyped and nine (5%) *DPYD* IM phenotypes returned; there were six heterozygous carriers of HapB3 (c.1236G>A, c.1129-5923C>G) and three heterozygous carriers of c.2846A>T. No variants were detected in the remaining 172 patients, and thus they were predicted to have the NM phenotype. Two of the nine IMs had prior exposure to systemic FP, although both experienced significant adverse effects warranting a dose reduction, and *DPYD* was ordered pretreatment in the remaining seven IMs.

**Table 1:** Demographics, clinical characteristics and *DPYD* ordering trends during pilot period.

Patient characteristic	
Sex	N (%)
Female	104 (51.7)
Male	97 (48.3)
Age (mean years ± SD)	62 ± 11
Race	N (%)
White or European American	154 (76.6)
Black or African American	24 (11.9)
Asian/Native Hawaiian/Pacific Islander	2 (1)
Native Hawaiian/Pacific Islander	2 (1)
Unknown or Other/Mixed Race	19 (9.5)
Ethnicity	N (%)
Non-Hispanic/Latino	177 (88)
Hispanic/Latino	18 (9)
Unknown	6 (3)
Primary Cancer Type	N (%)
Rectal	49 (24.4)
Colon	45 (22.4)
Pancreatic	44 (21.9)
Esophageal	19 (9.4)
Anal	9 (4.5)
Breast	7 (3.5)
Cecal	7 (3.5)
Gastric	7 (3.5)
Other*	14 (6.9)
DPYD Phenotype, Activity Score (AS)	N (%)
Normal Metabolizer, AS = 2	172 (95)
Intermediate Metabolizer, AS = 1.5	9 (5)
DPYD Genotype Orders	N (%)
Ordered pretreatment in FP naïve patient	180 (89.5)
Prior FP exposure	11 (5.5)
Ordered after chemotherapy start	5 (2.5)
Ordered same day as chemotherapy start	5 (2.5)
DPYD Sample Collection of Pretreatment Orders (n =180)	N (%)
Collected pretreatment	153 (85)
Collected after chemotherapy start	8 (4.4)
Collected same day as chemotherapy start	4 (2.2)
Not collected	15 (8.3)
DPYD Turnaround Time	Days
Sample collection to genotype result (Mean ± SD)	5.0 ± 2.7
Genotype result return prior to chemotherapy start^†^ (Median (Q_3_-Q_1_))	15 (28-7)

† When ordered and collected at least 1 day pretreatment. FP, Fluoropyrimidine. *Ampullary (1), Appendiceal (3), Bile duct (3), Duodenal (1), Gallbladder (2), Liver (1), Maxilla (1), Nasopharyngeal (1), Penile (1)

The mean time from sample collection to *DPYD* result return was 5.0 ± 2.7 days. *DPYD* genotype returned prior to chemotherapy initiation for 80.3% (114/142) of all patients who received a FP-containing regimen, but some of these patients had the test ordered and/or collected after already initiating therapy. Results returned prior to chemotherapy initiation for 93.2% (110/118) of those who had DNA samples collected pretreatment (Median days = 15, IQR 28-7). *DPYD* results returned the same day as starting chemotherapy for 4.2% (5/118) of patients with samples collected pretreatment and after chemotherapy initiation for only 2.5% (3/118) patients – all of whom were *DPYD* NMs.

### 3.2 Clinical decision support

A consult note with specific FP dosing guidance was placed in the medical record and routed to the treating oncologist for all nine *DPYD* IMs. Consult notes were completed 0–4 days after result return, and most (n=7) were completed within one day. All IMs scheduled to start a FP-containing regimen (n=7) had a consult note placed by a PGx pharmacist prior to chemotherapy initiation.

Pharmacist-facing OPAs fired 22 total times for the 4 individuals with the *DPYD* IM (AS = 1.5) phenotype who initiated a FP-containing regimen within the pilot period. Guidance recommending a 50% reduction in FP dose and reminder for upward titration in subsequent cycles were included in the OPA. Pharmacists were required to select one of the following acknowledgement reasons when responding to the OPA: “dose adjusted”, indicating the FP starting dose was reduced by 50% in accordance with the alert recommendation; “defer to discuss with prescriber,” indicating the pharmacist opted to clarify the dosing plan with the oncologist; “dose not adjusted per provider,” indicating the prescriber elected not to modify the dose despite the recommendation; or “other-see comment,” allowing pharmacists to document an alternative rationale not captured by the predefined options. Across the 22 alerts, pharmacists selected “dose adjusted” in 19 instances, “defer to discuss with prescriber” in 2 instances, and “dose not adjusted per provider” in 1 instance, with no alerts documented as “other-see comment”. The one instance in which “dose not adjusted per provider” was selected occurred in cycle 3 after upward dose titrations based on patient tolerance were performed. A 50% dose reduction was performed in all IMs who newly initiated a FP-containing regimen within the pilot period.

## 4 Discussion

### 4.1 Challenges – planning phase

Stakeholder engagement is commonly cited as a major contributor to successful PGx implementation (31), thus, we sought out to achieve this during the planning phase of *DPYD* test implementation. Several challenges were encountered (and overcome) during this time. First, some oncologists were concerned that preemptive lowering of FP doses might adversely affect efficacy of the chemotherapeutic regimen, particularly when given with (neo)adjuvant or curative intent. To address this clinically relevant concern, we provided recently published evidence indicating that reducing doses in *DPYD* IMs and PMs does not appear to impact treatment outcomes when subsequent upward dose titrations are used (20). Further, our recommendations encouraged the prescriber to consider dose titration in IMs who tolerate the initial reduced FP dose if lack of efficacy is a concern. Some oncologists pointed out that there were no universally accepted guidelines for how to adjust FP dosing in IMs and PMs. While this is true, we clarified that CPIC provides evidence-based preemptive FP dose reduction recommendations based on patient *DPYD* genotype.

There was some concern related to whether testing was worth the resources and effort given how rare predicted *DPYD* PMs are (1% or less). We addressed this by clarifying that testing would be clinically important in both *DPYD* IMs and PMs. The combined prevalence of PMs and IMs (~5-6%) is much higher than the prevalence of deleterious variants in other genes recommended by the NCCN for routine testing, such as Lynch syndrome genes (*MLH1*, *MSH2*, *MSH6*, *PMS2*, and *EPCAM*) and *CDH1* (32). Further, while piloting the implementation we covered the cost of the clinical PGx testing, allowing oncologists to focus on assessing the feasibility of testing in their practice at no cost to the patient.

Most patients are unfamiliar with PGx testing, but providing education generally increases interest and perceived value of performing PGx testing among patients (33). Limited data exist regarding the availability, quality, and effectiveness of patient education materials related to PGx-guided medication management. In general, the underlying concepts (e.g., purpose of testing, interpretation of results) can be difficult to communicate (34). Most available educational materials are developed by laboratories and focus on specific assays rather than broader, patient-centered guidance (35). Patients prefer customized reports tailored to their conditions and medications, using plain language and minimal medical jargon (30, 34). We developed patient-facing material using a unique, patient-centered approach designed to present information using a combination of visual, written, and audio methods.

### 4.2 Challenges – implementation phase

There were a number of early challenges faced because of the unique workflow within the cancer clinic. Our standard workflow for PGx implementation for other gene-drug pairs relies on medication ordering to trigger CDS alerts. However, this would not work in the cancer clinic for many reasons. First, chemotherapeutics were rarely ordered using the standard drug order. Instead, chemotherapy treatment plans serve as the structural framework for regimen selection in Epic. They are built in advance of the actual medication administration date, which forced us to re-evaluate trigger criteria from what is typically used in our standard OPA interruptive or inline alerts. Second, while chemotherapy decisions were made at least a week before the first dose was administered, the medication orders were not actually signed until just prior to administration. Thus, we could not develop CDS to fire off of signing the drug order. Pharmacist-facing OPAs described above were developed as a pragmatic solution to the absent medication ordering OPA infrastructure in oncology treatment plans. They ensured that the right person was notified of necessary dose adjustments at the right time, as the verifying pharmacist was responsible for confirming appropriate dosing based on updated clinical factors (e.g., weight). Inline warnings embedded in the treatment plan served as a reminder to oncologists at the time of initially setting up chemotherapy to order *DPYD* genotyping if appropriate but could not be relied on to consistently notify them of recommended dose reductions once the results were available.

When tests were ordered in the oncology clinic, we identified challenges with the logistics of collecting and delivering the sample to the molecular pathology laboratory. Buccal swab collection was done successfully for many patients, but it proved to be an imperfect method of ensuring same-day collection. The option for in-clinic buccal swab collection, done in an effort to maximize sample collection, deviated from the staff’s typical blood-draw process. There was no automatic notification to the nursing team that an order was placed, and completion of the sample collection relied entirely on the ordering provider personally notifying nursing staff that the test was needed. As such, some patients left clinic without a sample collected. The workflow was adjusted to include the option to collect blood samples, and some oncologists and their staff adopted this as their primary method of sample collection. This did not completely eliminate uncollected samples, however, and increased communication when *DPYD* orders were placed was encouraged. Samples for outstanding orders may have been collected during future routine lab, hence, the variability in collection timing among pretreatment orders that was observed.

Throughout the pilot period there was a need for continuous educational efforts due to turnover of oncologists, pharmacists, and nursing personnel. While orientation, onboarding and education caused short-term setbacks, it also made it clear that processes to support new hires and provide repetitive reminders for established staff were necessary for long-term sustainability. Adding PGx-specific clinical support information into tools already being opened routinely (e.g., clinic schedule, treatment plans) helped reduce the number of extra clicks needed to facilitate testing, a desire expressed by oncologists, but also helped integrate *DPYD* ordering into standard processes performed by new personnel.

Another challenge involved clinicians’ understanding of nuances when integrating *DPYD* results into practice beyond the initial dose reduction. For example, we discovered early in our implementation process that recommendations provided for the initial chemotherapy cycle did not carry forward to the next. The post-test inline warning in every cycle of the treatment plan reminding providers of the patient’s predicted *DPYD* metabolizer status and corresponding medication recommendations was added to address this issue (**Figure 4**).

This pilot was executed in a setting with a resource-rich academic infrastructure and experience implementing PGx in non-oncology clinical areas. However, this was our first dedicated effort to implement PGx-guided chemotherapy which, as described above, comes with a unique set of challenges. Lessons learned and successful strategies, therefore, are still relevant to institutions with a shorter history of PGx integration.

### 4.3 Takeaways and future directions

We successfully developed a feasible workflow in collaboration with the UF Health Cancer Institute that integrates *DPYD* genotyping into clinical care (**Figure 6**). Unique CDS tools were designed intentionally to integrate into oncology-specific processes. Although a small sample size, we observed that potentially life-saving dose adjustments, when necessary, were performed as a result of these with little manual intervention. Epic Systems Corporation is the largest individual electronic health record (EHR) vendor and is used in nearly half of all US hospitals systems. Thus, a large number of cancer institutes have the ability to replicate and benefit from descriptions of this infrastructure. The CDS strategies described herein rely on discrete result variables from either in-house or commercial laboratory testing. These processes may be replicated at institutions using third-party vendors for *DPYD* genotyping so long as results are entered into the health record discreetly. Additionally, the automated nature of these tools may be particularly beneficial for institutions without the personnel or bandwidth to manually screen patients or provide individual pharmacist consult notes.

**Figure 6:**
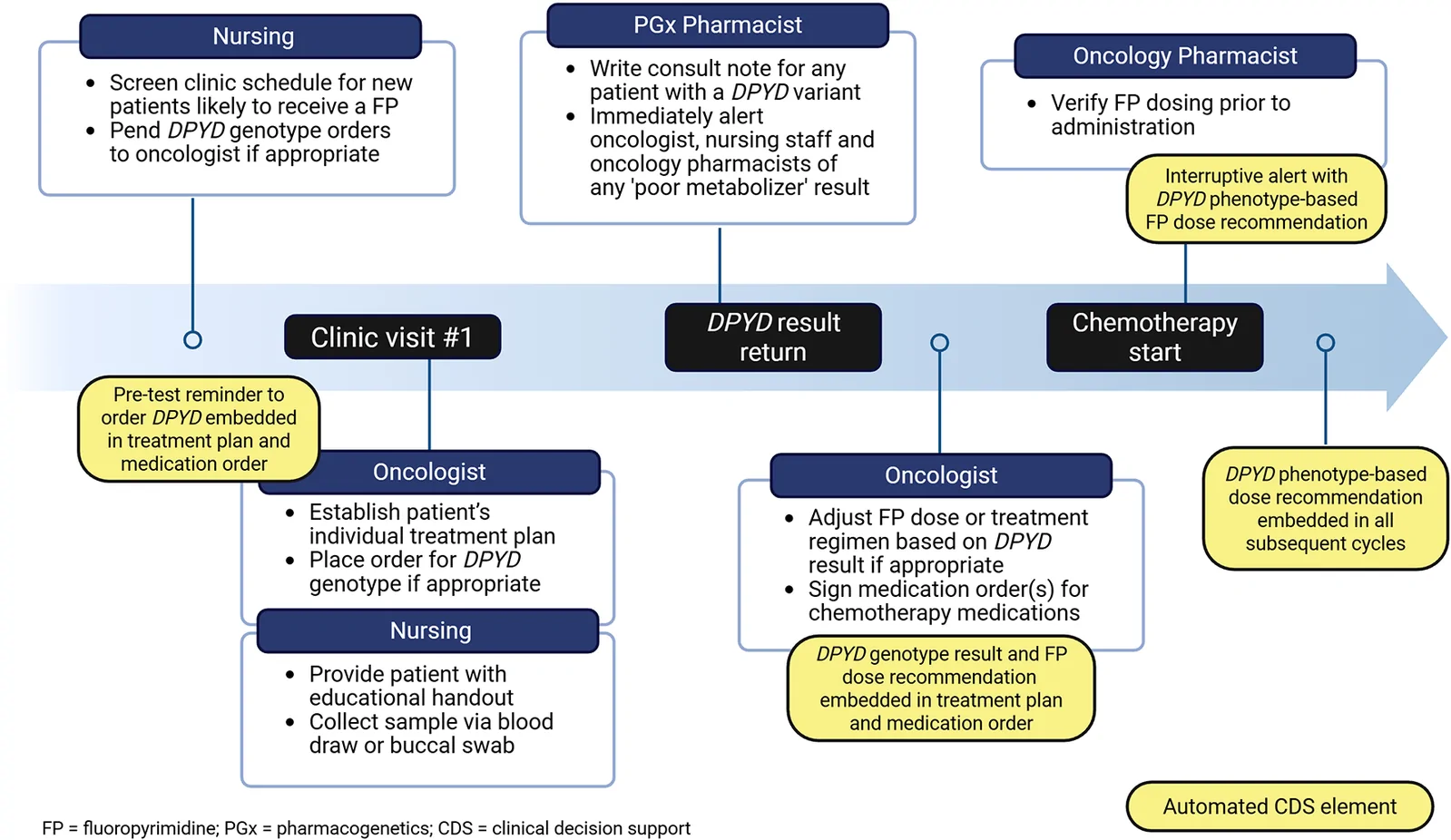
*DPYD* implementation workflow at the conclusion of the pilot period from time of pre-screening to the time of chemotherapy initiation. Responsible personnel are listed above each respective step (Created in BioRender. Norris, M. (2026) https://BioRender.com/kcf783o).

Having a dedicated hematology/oncology fellow review clinic schedules to identify patients for testing is no longer necessary as clinic personnel have become more comfortable with identifying patients for testing and placing orders. Over time, we have been able to transfer patient screenings to clinic nurses who screen and send unsigned *DPYD* orders to oncologists as part of their standard pre-visit workup process. We also achieved favorable turnaround times, with only 2.5% of tests resulting after the day of chemotherapy initiation when ordered and collected pretreatment. Implementation of *DPYD* genotyping into the clinical workflow in a manner that ensures results will be available in time to inform initial FP dosing is essential to reduce the risk of FP-related toxicity, but delaying the initiation of chemotherapy may nullify these benefits (36). The timeframe between a patient’s first clinic visit and initiation of chemotherapy was longer than initially estimated, decreasing turnaround time pressure and reducing the risk of delaying chemotherapy treatment at our institution.

Efficiently identifying and genotyping patients anticipated to receive FP-based chemotherapy regimen is key for achieving a turnaround time that does not delay chemotherapy. While we successfully achieved this in most cases, other institutions have reported significant delays in obtaining test results, ultimately affecting the ability to provide initial genotype-guided dosing (9). Continued efforts to further refine workflow integration to ensure all orders are placed and collected at appropriate time points will be the driving factor to minimize risk of treatment delays. A PGx pharmacist will continue to evaluate all results for clinical actionability, as this is our standard workflow for any PGx result in the UF Health system, however, there is now less need for assistance with patient identification and result interpretation.

Although toxicity and treatment outcomes were not formally analyzed as a part of this quality improvement pilot, dose reductions did not completely prevent adverse effects from occurring in variant carriers. In fact, we published a case report describing a c.2846A>T heterozygous carrier from our institution who experienced grade 3 neutropenia despite a 50% dose reduction (37). The possibility of undetected, decreased or no function variants in *DPYD* cannot be ruled out in any patient genotyped as a part of this pilot. We attempted to create an inclusive panel of *DPYD* variants relevant to various geographical populations; however, the genotyping assay was designed prior to the publication of the Association for Molecular Pathology’s (AMP) minimum set of recommended alleles for *DPYD* genotyping guidelines (38). One decreased function variant, c.868A>G, recommended by AMP as a Tier 1 “must-test” allele was not included. An updated assay is currently in development to add this missing variant.

At the completion of the pilot period, we transitioned to clinical billing of *DPYD* tests to insurance, and reimbursement data are being monitored. With the strengthening of test recommendations from both NCCN and the FDA, favorable reimbursement rates are expected. As other programs adopt *DPYD* testing in accordance with NCCN and FDA-approved guidance, our experience described herein is expected to provide important information to facilitate implementation strategies to overcome critical implementation barriers. Importantly, the pilot generated physician, nursing and pharmacy champions in the organization who are now part of our expansion of *DPYD* testing across UF Health cancer clinics.

## Data Availability

The original contributions presented in the study are included in the article/supplementary material. Further inquiries can be directed to the corresponding author.
